# Multimodal Magnetic Resonance Imaging in Diabetic Kidney Disease: From Pathophysiological Insights to Clinical Applications

**DOI:** 10.3390/diagnostics16111676

**Published:** 2026-05-29

**Authors:** Mengdan Ni, Bingcang Huang

**Affiliations:** 1 Public College of Medical Technology, University of Shanghai for Science and Technology, Shanghai 200093, China; nmd0208@sina.com; 2 Department of Radiology, Gongli Hospital, Pudong, Shanghai 200135, China

**Keywords:** diabetes mellitus, diabetic kidney disease, multimodal magnetic resonance imaging, renal dysfunction, renal tissue characteristics

## Abstract

Background: Diabetic kidney disease (DKD) is the leading cause of end-stage renal disease. Conventional clinical markers of renal function lack sufficient sensitivity for early diagnosis, whereas renal biopsy is unsuitable for routine monitoring because of its invasiveness. Objective: This narrative review aimed to evaluate recent advances in novel, non-invasive multimodal magnetic resonance imaging (MRI) biomarkers for the assessment of renal pathological alterations in DKD. Recent findings: Recent studies have demonstrated that multimodal MRI can non-invasively characterize several key pathological features of DKD, including renal hypoxia, microvascular dysfunction, ectopic fat deposition, and interstitial fibrosis. Furthermore, emerging evidence suggests that these imaging biomarkers may enhance risk stratification, monitor disease progression, and assess treatment efficacy, particularly in the presence of comorbidities and the advent of emerging therapies. Conclusions: Multimodal MRI shows considerable promise in translating advanced imaging biomarkers into clinical practice, facilitating the personalized management of DKD. However, future research must focus on establishing standardized imaging acquisition and analytical protocols, conducting prospective cohort studies to validate the association between imaging biomarkers and hard clinical endpoints, integrating artificial intelligence for automated analysis, and developing molecular imaging probes targeted at early disease pathways.

## 1. Introduction

Diabetic kidney disease (DKD) is not only one of the most severe microvascular complications of diabetes, but also the leading cause of end-stage renal disease (ESRD) globally [1,2]. Its incidence continues to rise in tandem with the growing diabetic population [1,3]. In patients with type 2 diabetes mellitus (T2DM), asymptomatic cardiac and renal structural and functional abnormalities are frequently observed and are strongly associated with an increased risk of renal failure [1,4,5]. Consequently, identifying pathophysiological changes in the kidney during the subclinical or normoalbuminuric stages is crucial for effectively intervening to prevent disease progression and avoid dialysis [6].

Clinically, DKD is primarily characterized by a sustained decline in estimated glomerular filtration rate (eGFR) and an elevation in the urinary albumin-to-creatinine ratio (UACR) [7]; however, these markers lack the sensitivity required to reflect early renal pathological changes [8]. Although renal biopsy remains the gold standard for diagnosis, its clinical utility for routine screening or longitudinal monitoring is limited by its invasiveness, risk of complications, and subjective differences in specimen interpretation [9]. Furthermore, conventional imaging modalities (such as ultrasound, IVP, and CT) are useful for evaluating renal function but have limited diagnostic efficacy in the early stages of the disease [10].

To address this clinical challenge, multimodal renal magnetic resonance imaging (MRI) has emerged as a focal point of research due to its ability to non-invasively and quantitatively assess renal oxygenation, perfusion, and structural and functional changes [11]. These techniques primarily include blood-oxygen-level-dependent (BOLD) imaging, arterial spin labeling (ASL), intravoxel incoherent motion (IVIM), diffusion-weighted imaging (DWI), diffusion tensor imaging (DTI), diffusion kurtosis imaging (DKI), and chemical shift encoding-based water–fat separation (Dixon) [12]. These modalities can directly detect early pathological processes in the kidney, such as hypoxia, iron deposition, microcirculatory impairment, and fibrosis, thereby providing essential imaging biomarkers that help elucidate the underlying pathophysiological mechanisms of DKD and support clinical decision-making (Table 1).

In this review, we summarize the impact of diabetes on renal structure and function, illustrating how MRI techniques elucidate the underlying pathophysiological mechanisms of renal dysfunction, improve risk stratification, and guide treatment strategies. Furthermore, we explore the role of MRI in evaluating the impact of major comorbidities and assessing novel therapeutic interventions.

## 2. Methods

This narrative literature review was conducted using the PubMed/Medline, Web of Science, and Google Scholar databases to identify studies investigating the application of multimodal MRI in DKD. Relevant articles published in English between 2000 and 2026 were reviewed. The primary search terms included “type 2 diabetes mellitus”, “diabetic kidney disease”, “magnetic resonance imaging”, “multiparametric MRI”, “functional MRI”, “blood oxygen level-dependent MRI”, “arterial spin labeling”, “diffusion-weighted imaging”, “diffusion tensor imaging”, “diffusion kurtosis imaging”, “intravoxel incoherent motion”, “$T_{1}$ mapping”, and “renal fibrosis”. Additional search terms were applied according to the focus of specific subsections, including “artificial intelligence”, “radiomics”, “machine learning”, “treatment response”, “drug efficacy”, “SGLT2 inhibitors”, and “GLP-1 receptor agonists”.

Our review focuses on the pathological basis, technical principles, clinical applications, advantages, and limitations of multimodal renal MRI in DKD, with particular emphasis on renal hypoxia, microvascular dysfunction, ectopic fat deposition, fibrosis, disease progression, therapeutic response assessment, and emerging artificial intelligence–assisted imaging analysis.

## 3. New Definition of DKD

Historically, the term “Diabetic Nephropathy” referred strictly to histopathological manifestations of glomerulosclerosis and fibrosis, as confirmed by biopsy. However, owing to the invasive nature of biopsies, current guidelines favor the clinical term “Diabetic Kidney Disease”. DKD is defined as the presence of persistently UACR ≥ 30 mg/g and/or eGFR < 60 mL/min/1.73 m^2^ for over three months in diabetic patients, in the absence of signs or symptoms of other primary kidney diseases. This definition reflects a move away from relying on biopsy findings and focusing solely on glomerular damage, toward a functional approach that covers both albuminuric and non-albuminuric forms of kidney disease [39,40].

## 4. Staging and Phenotyping

The staging and phenotyping of DKD are active and rapidly evolving fields of research. Currently, the clinical staging of DKD primarily relies on the KDIGO classification system, which categorizes disease severity along two orthogonal axes: eGFR and albuminuria [41]. This matrix provides prognostic risk categories ranging from low to very high, and it has been validated for predicting ESRD and cardiovascular events. However, eGFR has limitations during the early hyperfiltration phase, where renal function may appear preserved or even increased despite ongoing structural damage. Similarly, UACR is susceptible to interference from systemic hemodynamics, acute illness, exercise, and urinary tract infection, and it is predominantly a marker of glomerular permeability rather than tubulointerstitial injury [42]. Both markers fall short in accurately reflecting true renal microstructural damage.

Notably, the clinical presentation of DKD is highly heterogeneous. Recent epidemiological evidence indicates a rising incidence of non-albuminuric diabetic kidney disease (NA-DKD), characterized by a progressive decline in eGFR without preceding proteinuria. Population-based surveys suggest that 30–60% of T2DM patients with reduced eGFR may have normal albuminuria, with prevalence varying by ethnicity and region [43]. This phenotype may be predominantly driven by macrovascular atherosclerosis and tubulointerstitial fibrosis rather than by classic progressive glomerular injury, highlighting the limitations of conventional biomarkers for early diagnosis [44,45]. Importantly, although NA-DKD carries a lower risk of progression to end-stage kidney disease compared with albuminuric DKD, it remains associated with increased cardiovascular morbidity and mortality relative to patients without DKD, underscoring the need for distinct risk stratification strategies [43,46].

In this context, staging systems based solely on functional indicators are insufficient for precision medicine, driving the development of imaging-based phenotyping rooted in pathophysiological characteristics. Research indicates that the progression of DKD is influenced by a complex interplay of fibrosis, inflammation, hemodynamic disturbances, and hypoxia, with notable variability among individuals. Considering that imaging indicators based on a single modality lack specificity in capturing these intricate pathological processes, a comprehensive multiparametric MRI approach is essential. This methodology provides critical information that correlates with conventional biomarkers and uncovers underlying pathological details—concurrent hypoxia, inflammation, and fibrosis—within the diabetic environment. Consequently, it substantially improves DKD staging and phenotypic differentiation, potentially enabling the identification of high-risk subgroups before the onset of irreversible functional decline [13,47].

## 5. Pathophysiological Mechanisms

The pathophysiology of DKD is multifactorial and involves a complex interplay among metabolic dysregulation, hemodynamic alterations, inflammation, hypoxia, and progressive structural remodeling [48,49]. Chronic hyperglycemia and insulin resistance initiate a cascade of metabolic disturbances, including oxidative stress, mitochondrial dysfunction, advanced glycation end-product accumulation, and activation of pro-inflammatory and profibrotic signaling pathways [50,51]. In T2DM, hyperglycemia is considered the primary etiological factor driving DKD progression [49]. Concurrently, activation of the renin–angiotensin–aldosterone system (RAAS) and altered tubuloglomerular feedback contribute to glomerular hyperperfusion and hyperfiltration during the early stages of diabetes [48,49].

Increased glomerular filtration enhances tubular sodium and glucose reabsorption through sodium–glucose cotransporters in the proximal tubules, thereby markedly elevating renal oxygen consumption [52,53,54]. Reduced sodium chloride delivery to the macula densa further stimulates glomerular filtration, creating a maladaptive feedback loop that aggravates hyperfiltration [52,53,54]. At the same time, endothelial dysfunction and microvascular rarefaction impair oxygen delivery, leading to an imbalance between oxygen supply and demand within the renal parenchyma [52,54,55,56,57,58]. This mismatch is particularly pronounced in the renal medulla, which physiologically exists in a relatively hypoxic state because of its unique countercurrent exchange system and high metabolic activity. Persistent tissue hypoxia subsequently activates hypoxia-inducible pathways, inflammatory responses, and profibrotic signaling cascades, thereby promoting epithelial-to-mesenchymal transition, myofibroblast activation, and extracellular matrix deposition [59,60].

**Figure 1:**
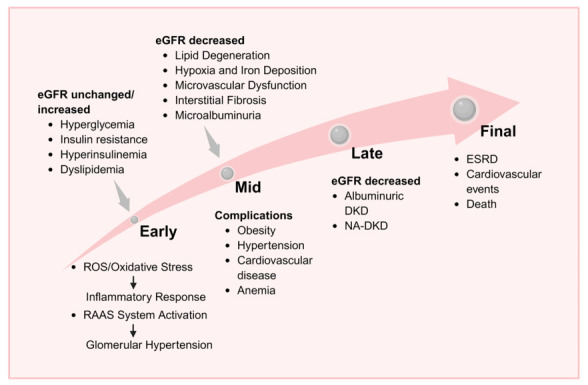
Progression of DKD and its underlying pathophysiological mechanisms [64,65]. The schematic depicts the temporal evolution of DKD across four key stages. In the early stage, despite eGFR remaining unchanged or transiently increased, initial metabolic perturbations (hyperglycemia, insulin resistance, and dyslipidemia) trigger ROS-induced oxidative stress, inflammatory responses, and RAAS activation, leading to glomerular hypertension. During the mid stage, systemic complications—including obesity, hypertension, cardiovascular disease, and anemia—emerge and further compound renal injury. As the disease advances to the late stage, a marked decline in eGFR occurs, driven by severe microenvironmental and structural alterations such as lipid degeneration, tissue hypoxia with iron deposition, microvascular dysfunction, and interstitial fibrosis. This functional decline clinically manifests as either classical albuminuric DKD or NA-DKD. Ultimately, the final stage culminates in ESRD, severe cardiovascular events, and death. NA-DKD, non-albuminuric DKD; ESRD, end-stage renal disease.

As renal fibrosis progresses, vascular rarefaction and tubular atrophy further aggravate hypoxia, forming a self-perpetuating vicious cycle of progressive renal oxygen deficiency [59,61,62]. Simultaneously, sustained intraglomerular hypertension and mechanical stress compromise the glomerular filtration barrier through podocyte injury, mesangial expansion, and endothelial dysfunction, ultimately leading to glomerulosclerosis, tubulointerstitial fibrosis, proteinuria, and declining renal function [59,63]. Fibrosis and tubular atrophy also contribute to macrostructural alterations in renal compartment volumes. These pathophysiological alterations collectively contribute to the heterogeneous clinical phenotypes of DKD, including albuminuric DKD and NA-DKD [64] (Figure 1).

## 6. Connecting MRI Biomarkers to Pathophysiological Changes in DKD

**Figure 2:**
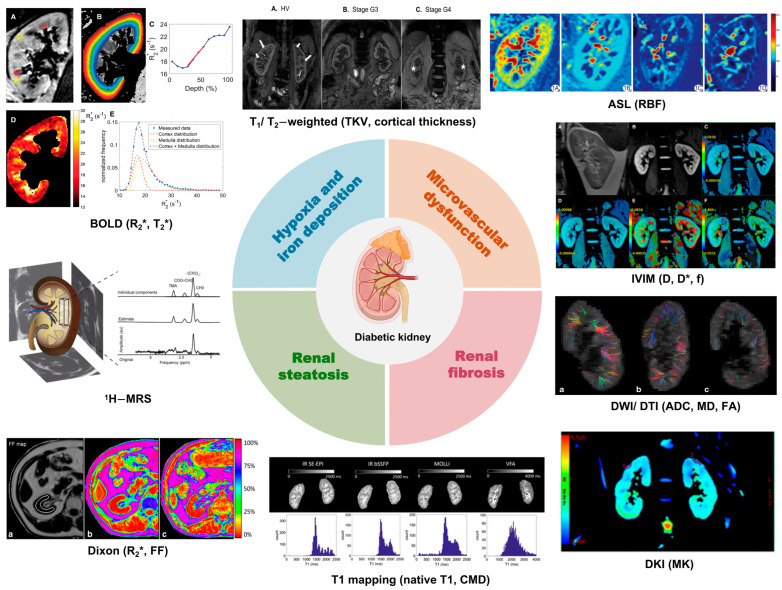
Multiparametric MRI biomarkers mapped to pathophysiological processes in DKD. The central schema assigns specific MRI techniques to four core pathological axes: (i) hypoxia and iron deposition assessed by BOLD; (ii) microvascular dysfunction evaluated by ASL and IVIM; (iii) renal steatosis quantified by Dixon and ^1^H-MRS; and (iv) interstitial fibrosis characterized by DWI, DTI, DKI, and $T_{1}$ mapping. Conventional $T_{1}$/$T_{2}$-weighted imaging provides macrostructural metrics. Several techniques provide a comprehensive assessment of overlapping mechanisms: Dixon captures both steatosis and iron deposition, IVIM distinctly separates microcapillary perfusion from pure tissue diffusion, and $T_{1}$ mapping reflects interstitial fibrosis, tissue edema, and loss of corticomedullary differentiation.

The pathophysiology of DKD involves interconnected processes, primarily including abnormal renal medullary metabolism and oxygenation, microvascular dysfunction and rarefaction, ectopic renal fat deposition, and interstitial fibrosis, leading to macroscopic structural and functional impairments. Multimodal MRI provides a unique perspective on these events, with specific techniques that directly reflect distinct pathological features (Figure 2).

### 6.1. Renal Tissue Hypoxia and Iron Deposition

BOLD-MRI exploits the paramagnetic effects of deoxyhemoglobin to shorten the transverse relaxation time constant ($T_{2}^{*}$), thereby reflecting tissue oxygenation status. This parameter can also be expressed as the apparent spin–spin relaxation rate ($R_{2}^{*}$) [14]. An elevated $R_{2}^{*}$ value indicates decreased tissue oxygenation, effectively revealing the early pathophysiological changes in DKD characterized by glomerular hyperfiltration and high metabolic load [66]. Studies confirm that, in T2DM patients, renal medullary $R_{2}^{*}$ correlates positively with fasting blood glucose and HbA1c levels. Furthermore, significantly elevated $R_{2}^{*}$ values are observed in the subclinical stage before eGFR decline, suggesting chronic hypoxia in the renal medulla [67]. Even in simple T2DM patients during the silent non-proteinuric phase, medullary $R_{2}^{*}$ and the medulla-to-cortex ratio is notably higher than in healthy controls, indicating that functional hypoxia precedes clinical signs such as microalbuminuria and can serve as an early warning signal [68].

However, BOLD-MRI yields heterogeneous results when assessing DKD progression and prognosis [13,65,69,70,71,72,73,74,75]. This is because the MRI signal reflects not only oxygenation, but is also influenced by the renal microenvironment [76]. As DKD progresses, anemia reduces hemoglobin (Hb) concentration, and microvascular rarefaction lowers the renal blood volume fraction (rBVF), both of which weaken the magnetic susceptibility effect and reduce the $R_{2}^{*}$ signal. Simultaneously, tubular atrophy and interstitial matrix accumulation alter the tubular volume fraction (TVF); since transverse relaxation time is highly sensitive to tissue hydration, this also changes $R_{2}^{*}$. These combined factors can offset hypoxia-induced $R_{2}^{*}$ elevations, leading to false negatives [77]. Thus, a combined assessment of TVF and rBVF, corrected for Hb levels, is necessary for accurately defining the role of BOLD-MRI.

Alongside oxygen metabolism, iron homeostasis imbalance and subsequent oxidative stress are critical mechanisms of T2DM-induced renal injury. Ren et al. combined BOLD and mDixon Quant techniques to quantitatively assess the kidney for both oxygen metabolism and iron deposition [35]. Using mDixon’s superior water–fat separation, they found that early T2DM patients exhibited both medullary hypoxia (elevated BOLD-$R_{2}^{*}$) and significant iron overload (elevated mDixon-$R_{2}^{*}$). ROC analysis showed that combining these metrics significantly outperformed single sequences. Translational research on routine clinical scans also shows that the kidney-to-muscle signal intensity ratio (KMR), calculated from standard $T_{2}$-weighted imaging, effectively reflects renal iron deposition in DKD [36]. As DKD progresses, paramagnetic iron deposition shortens $T_{2}$, causing a progressive decline in cortical and medullary KMR, which correlates with eGFR and cystatin C. In summary, local hypoxia under high-glucose conditions likely acts synergistically with ferroptosis-related iron deposition, providing highly specific imaging biomarkers for early DKD recognition.

### 6.2. Renal Microvascular Dysfunction

Renal microvascular rarefaction and endothelial dysfunction are crucial early features of DKD. These changes typically precede overt clinical proteinuria and irreversible fibrosis, making them key early warning indicators [78,79]. At the microcirculatory hemodynamic level, multimodal MRI reveals decreased perfusion capacity. ASL employs magnetically labeled arterial blood water as a completely endogenous tracer, with pseudo-continuous ASL being the most prevalent clinical application. By subtracting labeled and control images, quantitative renal blood flow (RBF) maps are produced without the need for exogenous contrast agents [16]. Using 3.0T ASL-MRI, studies show that RBF in the diabetic cortex is significantly lower than in healthy controls, even when eGFR remains normal. A progressive, segmental RBF decline is observed across advancing disease stages, with the posterior segment consistently identified as the most vulnerable region. Reduced RBF also inversely correlates with serum creatinine, triglycerides, and cystatin C [80,81]. These findings suggest that ASL may detect preclinical microvascular impairment before functional decompensation. While cortical perfusion demonstrates good reproducibility, medullary perfusion quantification remains challenging due to lower contrast-to-noise ratios, prolonged arterial transit times, and greater susceptibility to respiratory motion artifacts. Variability in labeling strategies, motion correction methods, and kinetic modeling assumptions also limits inter-study comparability [16]. In addition, most published studies have involved relatively small and heterogeneous cohorts, with inadequate control of physiological factors such as hydration status and circadian variation, thereby limiting the statistical power of subgroup analyses in advanced DKD [16,80,81]. Although consensus recommendations have been proposed to standardize renal ASL [82], thus promoting standardization of renal perfusion measurements and facilitating the comparability of results across scanners and in multi-center clinical studies, these guidelines remain largely expert-opinion based and await prospective multicenter validation as more data become available.

Complementing ASL’s macroscopic renal blood flow measurements, IVIM employs a biexponential model to separate pure molecular diffusion (D) from microcapillary perfusion, the latter quantified by the pseudo-diffusion coefficient ($D^{*}$) and perfusion fraction (f). In normoalbuminuric T2DM patients, reduced $D^{*}$ and f values reflect diminished capillary perfusion velocity and decreased functional microvascular density, thereby revealing microcirculatory impairment before the onset of albuminuria [83]. However, $D^{*}$ and f values exhibit lower reproducibility as they may reflect perfusion fluctuations across the cardiac cycle [84,85]. Furthermore, their individual diagnostic efficacy is inferior to that of the pure diffusion coefficient (D), likely due to measurement precision issues rather than fundamental biological mechanisms [86,87]. Additionally, b-value sampling schemes and fitting algorithms differ across studies, which will increase analytical variability and complicate the biological interpretation of IVIM metrics [88].

Multimodal MRI strategies have demonstrated superior diagnostic performance compared to single-sequence imaging. The combination of ASL and IVIM detects significant microcirculatory impairment during the silent normoalbuminuric phase, when urine protein levels remain normal [89]. Notably, the f value correlates strongly with RBF. Further combined analyses indicate that multi-parameter models can detect haemodynamic changes associated with microvascular sparsity with greater sensitivity to enhance diagnostic performance. Nevertheless, multimodal scanning inevitably increases clinical scan time and costs, as well as the complexity of analysis, thereby limiting its clinical application and validation.

### 6.3. Renal Steatosis and Perirenal Ectopic Fat Distribution

Ectopic fat deposition within the renal parenchyma is a hallmark of diabetic metabolic derangement and is significantly associated with increased risk of chronic kidney disease [90]. Multimodal MRI non-invasively evaluates harmful fat deposition, providing clues to the metabolic mechanisms driving fatty kidney disease [91,92]. Proton magnetic resonance spectroscopy (^1^H-MRS) exploits chemical shift differences to resolve lipid-specific resonances, enabling direct quantification of intracellular triglyceride content through the lipid-to-water peak area ratio [93]. However, respiratory motion sensitivity, prolonged acquisition times, complex spectral fitting, and susceptibility to magnetic field inhomogeneity limit its routine clinical application in the kidney [94]. In contrast, Dixon-based chemical shift encoding acquires multi-echo gradient-echo images. It exploits the ~3.5 ppm resonance frequency difference between water and fat protons, enabling pixel-wise signal decomposition and quantitative proton density fat fraction (PDFF) mapping. These high-resolution PDFF maps permit precise segmentation of the renal parenchyma, renal sinus, and perirenal fat [95]. Prospective cohorts show that the Dixon-quantified renal fat fraction (FF) is an independent predictor of renal deterioration in T2DM. High FF correlates with elevated UACR and reduced eGFR, offering superior risk stratification independent of visceral and subcutaneous fat [96]. Furthermore, elevated renal PDFF correlates positively with BOLD-$R_{2}^{*}$, suggesting lipotoxicity-induced oxidative stress exacerbates medullary hypoxia, jointly promoting DKD progression [97].

Despite its potential, several technical and methodological limitations hinder the clinical translation of Dixon-based renal fat quantification. Renal parenchymal fat content is intrinsically low; consequently, directly applying liver-optimized Dixon protocols to the kidney often yields unreliable cortical fat fractions, with values sometimes ranging erratically from below zero to over 10% [84]. Furthermore, the reproducibility of these measurements varies significantly by anatomical depot: while the quantification of renal sinus fat demonstrates acceptable repeatability, parenchymal PDFF remains less robust [84]. Standardization across different MRI vendors and post-processing algorithms is also lacking. Additionally, although two-point Dixon is widely available, it becomes unreliable in the setting of iron deposition because unmitigated $T_{2}$ signal decay confounds fat quantification; multi-echo Dixon mitigates this to some extent, yet its ability to grade mild steatosis may still be compromised [98]. Finally, the current evidence base predominantly consists of small, single-center, cross-sectional studies, and validated diagnostic thresholds for DKD have not yet been established.

MRI also reveals the pathogenic mechanisms of renal sinus adipose tissue (RSAT) and perirenal adipose tissue (PAT) [99]. Anatomically and functionally, these two adipose depots exhibit distinct characteristics: RSAT envelops the renal pelvis and vasculature, while PAT resides within the renal fascia [100]. Excessive accumulation of RSAT may physically compress the renal vessels and neural bundles, thereby impeding renal venous return, elevating renal interstitial hydrostatic pressure, and activating the RAAS [101]. Conversely, PAT exerts paracrine functions, secreting pro-inflammatory adipokines including leptin, resistin, and TNF-α. Given the absence of a fascial barrier between PAT and the renal cortex, these factors can directly permeate into the adjacent parenchyma, inciting inflammatory responses and fibrotic changes in renal tubular epithelial cells [56,102,103]. ASL and Dixon studies demonstrate that excessive perirenal and renal sinus fat deposition closely correlates with significantly reduced cortical and medullary RBF, confirming the link between physical compression and impaired perfusion early in DKD [104]. Despite this, no clinical imaging studies have reported on the inflammatory mechanisms mediated by PAT’s paracrine pro-inflammatory factors. Developing molecular probes targeting specific inflammatory factors or utilizing MRS to explore the relationship between fatty acid composition profiles and the local inflammatory microenvironment represent valuable directions for future research. Until then, comprehensive MRI assessment of intrarenal parenchymal lipids and renal sinus/perirenal fat, combined with liquid biopsy, remains one of the current approaches to elucidate the mechanisms of fat-mediated nephrotoxicity. This not only aids in identifying high-risk phenotypes during the silent phase before the onset of proteinuria but also holds promise for providing crucial imaging biomarkers to evaluate the therapeutic efficacy of interventions targeting lipid metabolism regulation [96].

### 6.4. Renal Fibrosis

Tubulointerstitial fibrosis is not only the final common pathway for the progression of DKD to ESRD, but also the key pathological basis determining renal functional prognosis. During renal fibrosis, the abnormal deposition of collagen and other extracellular matrix components significantly narrows the intercellular spaces and increases tissue density, thereby restricting the diffusion of water molecules [105,106]. DWI applies diffusion-sensitizing gradients at multiple b-values to quantify restricted diffusion, yielding the apparent diffusion coefficient (ADC) as a quantitative index of interstitial structural remodeling and microenvironmental alterations in DKD [107]. Consequently, ADC serves as a noninvasive biomarker for the early detection of renal injury and the monitoring of fibrotic progression. Clinical studies indicate that renal cortical ADC values are higher than those in the medulla among patients with T2DM. Cortical ADC correlates positively with eGFR and negatively with laboratory markers such as creatinine and cystatin C. Thus, it serves as an imaging indicator of renal functional status. Additionally, medullary FA values correlate positively with eGFR and are lower in diabetic patients than in healthy controls, indicating higher sensitivity in detecting early diabetic renal injury [108].

However, in the highly vascularized kidney, ADC is inherently confounded by concurrent micro perfusion and tubular fluid flow, limiting its pathological specificity for interstitial fibrosis. IVIM overcomes this by isolating the perfusion-corrected pure diffusion coefficient from hemodynamic signal contamination [109]. Compared with ADC, D exhibits higher specificity and stability in assessing DKD-related renal interstitial fibrosis and proteinuria. Feng et al. confirmed that the corrected D value more accurately reflects the degree of fibrosis and correlates negatively with the albumin-to-creatinine ratio [87]. Some studies further compared the diagnostic efficacy of IVIM and DTI. They found that the D value showed high sensitivity for identifying renal injury in normoalbuminuric T2DM patients, and its pathological specificity was also significantly superior to that of DTI parameters [83]. However, for the highly ordered radial anatomical structure of the renal medulla, DTI still retains unique structural imaging advantages. DTI applies diffusion encoding along at least six non-collinear directions to construct a second-order diffusion tensor. Quantifying fractional anisotropy (FA), it captures the loss of structural directionality caused by tubular atrophy and interstitial fibrosis. Imaging evidence shows that medullary FA values decline significantly in the early stages of DKD—even when eGFR remains normal—and before any cortical changes become apparent. These values progressively decrease as renal injury worsens, closely correlating with tubular injury and interstitial fibrosis scores [69,108,110].

As tissue heterogeneity increases during fibrosis, water molecule diffusion deviates from a Gaussian distribution. Diffusion kurtosis imaging (DKI) addresses this by acquiring data at high b-values to quantify non-Gaussian diffusion through the kurtosis metric, which captures the microstructural heterogeneity imparted by cross-linked fibrillar matrices. Mean kurtosis (MK) correlates positively with pathology-confirmed IFTA scores and the proportion of cortical fibrosis, showing better diagnostic efficacy than conventional DWI parameters [24,111]. Compared with conventional DWI parameters, the K value is more sensitive to non-Gaussian diffusion arising from cross-linked fibrillar matrices, providing unique advantages for evaluating the complex microenvironment during DKD fibrosis. Because DKI necessitates high b-values to capture non-Gaussian diffusion, the consequent reduction in SNR and extended acquisition times exacerbate its vulnerability to respiratory motion artifacts and magnetic field inhomogeneities. Furthermore, early fibrotic changes in DKD may be obscured by inter-individual variability. As a result, its current application is predominantly confined to diabetic rat models, where various DKI-derived parameters have established robust correlations with histological fibrosis grades [112]. Addressing the existing hurdles related to scan time, data processing, and standardization, as well as clinical validation, will be essential for broadening its future translational application.

Pure diffusion imaging reflects the restricted movement of water molecules. In addition, magnetization transfer ratio (MTR) quantifies the exchange of protons between free water and water bound to macromolecules in the extracellular matrix. It therefore reflects extracellular matrix deposition and has been applied to assess renal fibrosis. While MTR has been extensively studied in animal models with mixed results [113,114], evidence in human DKD remains limited. In a prospective cohort, MTR showed reasonable repeatability, yet its ICC values were modest to poor. Moreover, its correlation with mGFR was weak, and after correction for multiple comparisons, it showed no significant correlation with other MRI, plasma, or urine biomarkers [13].

Additionally, native $T_{1}$ mapping quantifies the longitudinal relaxation time of water molecules using inversion-recovery or variable-flip-angle spoiled gradient-echo sequences, without the need for exogenous contrast administration. Rising $T_{1}$ values typically reflect pathological changes such as edema (e.g., from inflammation-related water accumulation) and expansion of the interstitial space (e.g., due to fibrosis) [115]. Recent prospective studies have found that native $T_{1}$ values in the renal cortex and medulla of patients with DKD increase with disease progression. This is closely associated with the expansion of the extracellular matrix and the increase in water content during fibrosis, which prolongs the longitudinal relaxation time [116]. Although $T_{1}$ exhibits high specificity in identifying patients with advanced DKD, but it is not sensitive enough to early DKD. In fact, a key challenge in renal imaging is differentiating inflammatory edema from fibrosis, as both can elevate $T_{1}$ values. This limitation is precisely why a multi-parametric approach is necessary. The DTI and DKI parameters provide crucial context: pure inflammation or edema would likely present with high $T_{1}$ and increased DTI-MD, with a low or normal DKI-MK. In contrast, fibrosis presents as high $T_{1}$, decreased DTI-MD, and increased DKI-MK. Integrating native $T_{1}$, DTI, and DKI into a combined model enabled complementary imaging across the three levels of matrix composition, structural arrangement, and microenvironmental complexity [116]. This model achieved early DKD diagnostic efficacy superior to that of any single modality, indicating that fibrosis assessment is shifting from single indicators to comprehensive multiparametric characterization.

## 7. Assessment of the Effect of Comorbidities on Diabetic Kidney by Multimodal MRI

In addition to hyperglycemia-mediated mechanisms, comorbidities, including hypertension, obesity, cardiovascular disease, and anemia, may modulate renal injury in T2DM through pathways involving hypoxia, inflammation, and hemodynamic stress. The following summarizes preliminary evidence on the use of multimodal MRI to characterize these superimposed effects.

### 7.1. Hypertension

Hypertension is one of the most common comorbidities in T2DM patients, affecting more than two-thirds of this population [117]. Multiple clinical and basic studies confirm that hypertension is a crucial independent risk factor promoting the onset and progression of DKD [118,119,120]. Hypertension induces and exacerbates kidney disease by increasing intraglomerular pressure, damaging endothelial cells, and increasing sympathetic activity [121,122,123]; its coexistence with diabetes also significantly increases target organ damage [124]. BOLD-MRI studies found that in T2DM patients with microalbuminuria and/or hypertension, elevated systolic and diastolic blood pressure closely correlated with renal medullary hypoxia, whereas elevated blood glucose and glycated hemoglobin levels significantly correlated with renal cortical hypoxia. Nonetheless, after one month of antihypertensive treatment with traditional RAS blockers, neither cortical nor medullary BOLD-$R_{2}^{*}$ signals showed significant improvement [125]. Therefore, the specific mechanisms of action of hypertension or antihypertensive medications on DKD kidney tissue, as well as the identification of appropriate imaging biomarkers, require further exploration.

### 7.2. Obesity

Obesity and diabetes are collectively referred to as “Diabesity,” which is typically accompanied by the deposition of visceral, perirenal, and intrarenal parenchymal fat, serving as an independent risk factor for DKD [126,127]. Compared with systemic obesity, ectopic fat deposition—including visceral adipose tissue (VAT), PAT, and RSAT—exerts a more direct destructive effect in promoting renal microvascular injury. They cause structural changes in the kidney through lipotoxicity and the release of pro-inflammatory cytokines. The abnormal accumulation of renal sinus fat can also physically compress renal blood and lymphatic vessels, leading to local renal hemodynamic abnormalities and hyperfiltration states [100]. A multiparametric MRI study found that in prediabetic populations who have not yet developed diabetes, renal sinus fat volume already showed a significant increase and was independently correlated with systemic visceral fat volume and glucose metabolism indicators [128]. As the disease progresses to the early DKD stage, assessments utilizing Dixon imaging combined with DTI further revealed that T2DM patients with microalbuminuria had significantly higher renal fat fractions than normoalbuminuric and healthy control groups. Moreover, the elevated renal fat fraction showed a significant negative correlation with the FA value, demonstrating the synchronicity of ectopic lipid deposition and early renal damage [34].

### 7.3. Cardiovascular Disease

In T2DM, the cardiorenal axis constitutes a high-risk clinical paradigm: cardiovascular disease has become the leading cause of death in end-stage renal disease. Epidemiological data show that the excess mortality rate in diabetic patients is largely confined to those with concurrent kidney disease. This excess is mainly due to their high cardiovascular disease burden [129]. The heart and kidney interact in a mutually causative way, known as cardiorenal syndrome (CRS). Chronic cardiac insufficiency leads to systemic venous congestion and reduced cardiac output. These conditions cause renal hemodynamic disturbances and persistent tissue ischemia and hypoxia. Reduced cardiac pumping also overactivates the RAAS and sympathetic nervous systems. Coupled with the release of advanced glycation end products and systemic inflammatory mediators, this process further aggravates renal endothelial dysfunction and microvascular rarefaction. Ultimately, this leads to renal interstitial fibrosis [130,131]. Jin et al. used IVIM technology to evaluate patients with type 2 cardiorenal syndrome. They confirmed that compared to patients with simple chronic heart failure and healthy controls, the D, $D^{*}$ and f of the kidneys in type 2 CRS patients were significantly decreased. This reflects the severe impairment of renal microcirculatory perfusion caused by heart failure and provides imaging evidence for secondary early diffuse renal interstitial fibrosis and cellular edema [132].

### 7.4. Anemia

Anemia is a common comorbidity in diabetic patients [133]. Compared with anemia in non-diabetic CKD, anemia in DKD patients occurs earlier and is more severe [134,135]. The primary mechanisms include chronic inflammation induced by hyperglycemia, decreased EPO release due to diabetic autonomic neuropathy, and damage to EPO-producing cells caused by renal interstitial fibrosis [136,137,138]. Anemia can cause renal tissue hypoxia, thereby accelerating renal fibrosis. Concurrently, hypoxia stimulates cells to release cytokines and inflammatory mediators. This enhances sympathetic activity, which reduces renal blood flow and lowers GFR, ultimately exacerbating renal damage. Clinical cohort data indicate that a decline in Hb levels is an independent risk factor for rapid eGFR decline and adverse outcomes in DKD patients [139,140]. On MRI, medullary BOLD-$R_{2}^{*}$ values are significantly elevated, whereas cortical IVIM-D values decreased; both correlate with eGFR [139]. These findings suggest that long-standing hyperglycemia in DKD is associated with renal tissue hypoxia, diffuse interstitial fibrosis, and capillary damage. Importantly, the combined application of the $R_{2}^{*}$ value and the D value demonstrates greater diagnostic efficacy in predicting adverse outcomes in DKD than a single indicator. However, although anemia can be viewed as a marker of renal injury, its direct role in the onset or progression of diabetic complications has yet to be clearly elucidated. Future large-sample, multi-center studies are needed to explore the true impact of anemia or anti-anemia drug treatments on renal microstructure and function.

Furthermore, several other conditions prevalent in T2DM, including obstructive sleep apnea (OSA), hyperuricemia, and hyperlipidemia, may independently influence renal MRI signals. OSA, which is highly prevalent in diabetic populations, causes nocturnal intermittent hypoxia and sympathetic activation that may alter renal oxygenation and hemodynamics [141,142,143]; however, direct evidence linking OSA severity to altered renal BOLD or perfusion MRI in DKD is currently lacking. Hyperuricemia has been associated with restricted water diffusion, microstructural disorganization, and altered oxygenation as assessed by multiparametric MRI [144]. However, these findings derive from non-diabetic hyperuricemic cohorts, and their relevance to DKD remains uncertain. Hyperlipidemia, as a core metabolic disturbance, promotes lipotoxicity and endothelial dysfunction, but isolated effects on renal MRI phenotypes have not been specifically characterized. It should be emphasized that existing studies remain subject to notable methodological limitations: the evidence primarily derives from small, single-center observational cohorts with considerable heterogeneity in imaging protocols and patient phenotypes. Current data are insufficient to definitively distinguish whether the observed multimodal MRI alterations reflect independent pathophysiological effects of individual comorbidities or represent nonspecific features of generalized DKD progression. Consequently, the robustness and clinical significance of these findings await validation in prospective, large-scale, multicenter studies.

## 8. MRI-Guided Treatment Strategies: Traditional Drugs and Emerging Therapies

Sodium–glucose cotransporter-2 (SGLT2) inhibitors, initially developed as hypoglycemic agents, have now been proven in large clinical trials to significantly delay the progression of DKD [145] and effectively reduce the risk of renal failure [146]. Functional magnetic resonance imaging reveals that their renal protective effects may stem from two mechanisms: regulation of systemic sodium homeostasis and reduction in local metabolic burden. First, Karg et al. used ^23^Na-MRI to confirm that 6 weeks of dapagliflozin treatment significantly reduced tissue sodium content in the skin and muscle of T2DM patients; this is considered an early imaging marker for correcting systemic water-sodium retention and improving microcirculation [147]. Second, locally in the kidney, SGLT2 inhibitors significantly reduce the energy-consuming burden of the Na^+^/K^+^-ATPase by blocking proximal tubular sodium reabsorption, thereby alleviating corticomedullary hypoxia driven by hyperfiltration [52,148]. However, whether this functional improvement translates into structural repair remains controversial.

Additionally, glucagon-like peptide-1 receptor agonists (GLP-1 RAs) also exhibit renoprotective effects. For example, semaglutide upregulates β-Klotho gene in renal tubular epithelial cells. This activates the AMPK/SIRT1/NRF2 axis, thereby inhibiting ferroptosis and blocking the downstream NF-κB inflammatory pathway and TGF-β1/Smad fibrotic cascade [149]. At the imaging level, multiparametric MRI has revealed the effects of this single-drug therapy on renal hemodynamics, oxygenation, and microstructure. ASL shows significantly improved RBF after 28 weeks. BOLD-MRI demonstrates decreased cortical and medullary $R_{2}^{*}$ values, suggesting alleviation of medullary hypoxia; this parallels the decline of ferroptosis markers and recovery of GSH levels. DTI shows significant changes in cortical FA values, and modified Dixon imaging reveals significant changes in FF. These results indicate that GLP-1 RAs may improve renal microstructural integrity by mitigating interstitial edema, regulating lipid deposition, and modulating extracellular matrix remodeling [149]. However, Vernstrøm et al. found that semaglutide-induced reductions in cortical ADC and the narrowing of cortico-medullary ADC differences showed no significant correlation with changes in GFR, UACR, or inflammatory markers [150]. It is hypothesized that DWI changes may follow a trajectory independent of traditional renal function indicators, and their validity as biomarkers of treatment response remains to be validated longitudinally. Therefore, further establishing the correspondence between imaging and pathology to clarify whether these early changes foreshadow long-term hard-endpoint benefits is crucial.

As a novel class of RAAS-blocking agents, angiotensin receptor–neprilysin inhibitors have been shown to possess natriuretic, diuretic, and vasodilatory effects. BOLD-MRI studies report that sacubitril/valsartan significantly reduced renal medullary $R_{2}^{*}$ values in T2DM patients [151]. This improvement in oxygenation was closely associated with reduced urinary albumin, suggesting that the drug may improve renal hemodynamics and metabolic supply-demand balance. However, imaging evidence of improvement in renal tissue hypoxia with traditional blocking drugs remains limited and inconsistent [125]. These discrepancies may stem from differences in sample sizes, study populations, or drug characteristics. Further multicenter, large-sample studies are needed to understand the imaging improvements in renal structure and function with different RAAS-blocking drugs.

Regarding cutting-edge cellular immunotherapies, multimodal MRI can detect the reversal of renal structural damage. A clinical trial using autologous dendritic cells to treat DKD innovatively utilized DTI as an endpoint for therapeutic efficacy. Results indicated that post-treatment, patients’ fractional FA values significantly rebounded and were highly correlated with the degree of downregulation of serum inflammatory factors. Thus, compared to traditional drugs that primarily regulate hemodynamics, therapies targeting the immune microenvironment may lead to more profound microstructural remodeling [152].

In summary, although multimodal MRI reflects the protective effects of various therapeutic modalities on DKD (Table 2), current data predominantly focus on indirect markers of hemodynamics or metabolic function, and some results are contradictory within heterogeneous populations. To verify whether these therapies can reverse the characteristic pathological manifestations of DKD, designing rigorous multimodal MRI studies is an indispensable and vital next step.

## 9. Correlation of MRI Parameters with Circulating Biomarkers of Fibrosis and Inflammation

Given the central roles of fibrosis and inflammation in the pathogenesis of DKD, circulating biomarkers targeting these processes have attracted significant attention. Combining these molecular markers with multimodal MRI parameters enables multidimensional assessment, linking imaging phenotypes with molecular pathways in DKD. However, although many biomarkers have been shown to closely correlate with disease progression and prognosis in DKD [153], studies correlating them with MRI biomarkers remain scarce, and results are inconsistent.

In cross-sectional studies, inflammatory factors exhibit different correlations with MRI parameters. The DWI-ADC value, which reflects renal cortical fibrosis, correlates negatively with serum IL-6 and TNF-α. In contrast, the BOLD-$R_{2}^{*}$ value, which reflects tissue hypoxia, shows no independent correlation with these cytokines [154]. However, this evidence derives from a mixed CKD cohort rather than from DKD-specific populations. In longitudinal intervention studies, MRI parameters have shown the potential to reflect molecular pathological changes. A clinical trial of immunomodulation for DKD confirmed that FA not only sensitively captures microstructural remodeling post-treatment, but also that the degree of improvement closely aligns with the decline in serum levels of TGF-β1 and IL-6. This suggests that DTI can serve as a surrogate imaging endpoint to monitor the improvement of fibrosis mediated by anti-inflammatory molecules [152].

Beyond these, circulating inflammatory and fibrotic markers that have been associated with DKD progression and kidney failure include soluble tumor necrosis factor receptors 1 and 2 (TNFR-1 and TNFR-2), chitinase-3-like 1 (YKL-40), soluble urokinase-type plasminogen activator receptor (suPAR), and monocyte chemotactic protein-1 (MCP-1) [153]. Among these, TNFR-1 and TNFR-2 particularly reflect activation of the TNF pathway, which is involved in endothelial injury and glomerular damage, and they serve as important predictors of DKD progression. Theoretically, these circulating markers may correlate with MRI parameters reflecting renal microstructural damage and hypoxia, but this correlation has not been examined in prospective DKD cohorts. More recently, emerging fibrosis-specific biomarkers, such as procollagen type III N-terminal propeptide (PRO-C3) and matrix metalloproteinase-generated collagen degradation fragments (C1M and C3M), have shown potential in reflecting extracellular matrix remodeling in both diabetic and non-diabetic CKD [155,156]. Among these, serum C3M has been associated with inflammatory markers and with the risk of CKD progression in type 2 diabetic cohorts [157]. Compared with traditional cytokines, these collagen metabolism markers may more directly reflect ECM turnover and thus hold promise for greater specificity in quantifying tubulointerstitial fibrosis [158]. Although their specific relationship with MRI markers remains unknown, it will be a valuable direction for future research.

In summary, the combined assessment of MRI parameters and circulating biomarkers indicates that the diagnostic paradigm for DKD is shifting toward an integrated, multiparametric approach. Future research should focus on validating specific biomarker panels to enhance the detection of early occult lesions, optimize risk stratification, track disease progression, and evaluate the efficacy of targeted therapies through their synergistic application with renal magnetic resonance phenotyping.

## 10. MRI-Based Artificial Intelligence in DKD Risk Assessment

With the increasing integration of artificial intelligence and medical imaging, intelligent analysis based on multimodal magnetic resonance imaging is emerging as a promising approach for DKD risk stratification. For example, radiomics and machine learning can extract high-dimensional micro-pathological features invisible to the human eye from MRI data, providing non-invasive, quantitative, objective bases for the early identification, staging, and prognostic assessment of DKD. Nevertheless, their diagnostic and prognostic significance relative to traditional clinical and imaging biomarkers necessitates rigorous, prospective validation across diverse populations.

Recent preliminary studies suggest that radiomics combined with functional MRI may improve the diagnostic performance for DKD in selected cohorts. Yang et al. [159] prospectively evaluated a radiomics model derived from DTI and DKI in 163 patients with type 2 diabetes mellitus from a single center. The combined radiomics signature achieved an area under the receiver operating characteristic curve (AUC) of 0.918 in the internal validation cohort for discriminating DKD from T2DM without kidney disease. While these data are encouraging, the study was limited by a single-center design, a modest sample size, and the use of a complex diffusion spectrum imaging protocol that may be difficult to standardize across vendors and field strengths. Furthermore, although MK performed best in automated renal parenchyma segmentation, the generalizability of this deep learning–based segmentation to external datasets remains unproven. Additionally, Ma et al. developed an ASL based model in a single-center cohort of 163 participants. Using the least absolute shrinkage and selection operator regression, the investigators screened eight texture features from an initial set of 367, achieving an AUC of 0.865 for detecting diabetic renal injury across different stages [160]. However, the combined radiomics-clinical model achieved a lower AUC (0.734), suggesting limited discriminatory value of texture features beyond conventional variables. Furthermore, ASL perfusion measurements remain technically challenging owing to sensitivity to free-breathing artifacts, hematocrit levels, and antihypertensive medications.

Beyond functional imaging applications, the utility of routine anatomical sequences has also been explored using AI. Majos et al. applied deep learning algorithms to texture analysis of conventional T1WI, comparing multiple neural network architectures, including AlexNet, ResNet-50, and Vision Transformer. They found that the ResNet-50 model performed best in distinguishing chronic kidney disease stages, achieving a balanced accuracy of 93.1%, surpassing other models. These findings suggest that deep learning may capture texture patterns related to renal fibrosis and atrophy from routine anatomical images [161]. However, features derived from $T_{1}$-dependent images are inherently sensitive to scanner model, protocol settings, and reconstruction algorithms, which makes the reproducibility remain uncertain.

Given the inherent limitations of single modalities, the multimodal fusion of imaging, clinical data, and omics has been proposed as a strategy to enhance DKD risk assessment. Chen et al. extracted texture features from multimodal MRI (including T1WI, T2WI, DTI, and BOLD sequences) and evaluated the classification performance of five machine learning algorithms. The multimodal random forest model achieved an AUC of 0.91 for distinguishing early from moderate-to-advanced DKD in the training cohort, and an AUC of 0.88 in a small biopsy-subgroup for assessing interstitial fibrosis severity. These preliminary findings suggest that machine learning integrating multimodal imaging may capture information on renal functional decline and histological damage, though this hypothesis requires validation in larger, prospective cohorts [162]. It should also be noted that the AUC decline between the training and testing cohorts for non-severe renal impairment indicates overfitting, and manual ROI delineation and the lack of standardized texture extraction pipelines across institutions further constrain reproducibility. In a prospective single-center study, Shao et al. constructed a multimodal predictive model. It integrated MRI radiomics (T2WI, BOLD, and DTI), serum and urine biomarkers, and clinical indicators. The model was trained on 172 participants for DKD classification and 117 participants for prognostic analysis. The multimodal model increased the diagnostic AUC from 0.833 to 0.923 for DKD classification, and prognostic models reported AUCs of 0.975 and 0.932 for 2-year and 3-year progression risk, respectively [163]. While these performance metrics appear favorable, they should be interpreted cautiously. The prognostic cohort had a short median follow-up of only 1.21 years, and the study lacked external validation. Furthermore, statistically significant differences in baseline glycated hemoglobin between the training and testing sets may have yielded overoptimistic performance estimates, while the high-dimensional feature space relative to the modest sample size increases the risk of overfitting. Consequently, whether such multimodal models can reliably track dynamic disease trajectories and capture early signals of progression in DKD remains to be determined.

Although the joint application of AI and MRI has shown broad prospects for DKD risk assessment, current models still face challenges related to data heterogeneity and a lack of external validation. While machine learning models outperform traditional logistic regression models in prediction accuracy, most studies remain confined to single-center, small-sample cohorts and lack large-scale cross-ethnic and cross-device validation, which limits the generalization ability of the models [164]. Future efforts should focus on establishing large-scale, high-quality, multi-center imaging databases to validate the robustness and generalizability of radiomic signatures. Concurrently, there should be a shift from cross-sectional diagnosis to longitudinal prediction of disease trajectories, to track the dynamic evolution of imaging features over time and capture early signals of disease progression [165].

## 11. Discussion

Multimodal MRI has emerged as a promising tool for non-invasive characterization of the structural, functional, and microstructural alterations associated with DKD. Current evidence suggests that these techniques may provide complementary information beyond conventional clinical biomarkers, particularly for detecting early renal dysfunction, evaluating disease heterogeneity, and monitoring therapeutic response.

From our perspective, one of the major future directions in renal MRI research is the transition from single-parameter assessment toward integrated multiparametric imaging frameworks. DKD is a highly heterogeneous disease involving intertwined alterations in oxygenation, perfusion, tubular injury, inflammation, fibrosis, and ectopic lipid deposition. Consequently, individual MRI biomarkers may only reflect isolated aspects of renal pathophysiology and may not adequately capture disease complexity when interpreted independently. Multiparametric MRI combining functional, diffusion-based, perfusion-sensitive, and fat-quantification techniques may therefore provide a more comprehensive characterization of renal injury and improve individualized risk stratification. Moreover, integrating imaging biomarkers with clinical and laboratory data through artificial intelligence–based analytical frameworks may further enhance the predictive and translational value of renal MRI in precision nephrology.

Nevertheless, several important challenges remain before these techniques can move from research settings into routine clinical practice. First, individual MRI biomarkers—including BOLD, ASL, IVIM, Dixon, DKI, and native $T_{1}$ mapping—lack of harmonized acquisition protocols and validated post-processing pipelines [25,166]. Acquisition sequences and parameter settings differ substantially across MRI vendors and field strengths (1.5 T versus 3 T), leading to considerable inter-scanner and inter-center variability in quantitative metrics such as $T_{1}$, $T_{2}^{*}$, and ADC values [167,168]. Second, normative reference ranges for these parameters remain undefined in both healthy populations and DKD patients, complicating the establishment of clinically actionable thresholds [25,169]. Third, image quality is further compromised by respiratory, cardiac, and bowel motion artifacts, which are particularly challenging in patients with limited breath-holding capacity or advanced disease [169,170]. Fourth, the existing literature is dominated by single-center, cross-sectional studies with modest sample sizes, leaving the prognostic value of these biomarkers against hard renal endpoints largely unproven [171]. Furthermore, temporal and economic constraints further complicate clinical translation. Comprehensive multiparametric renal MRI protocols are time-consuming, often requiring prolonged breath-holds or multiple repeat scans, which reduce patient tolerance and throughput in clinical workflows. The associated costs, including prolonged scanner time, specialized sequences, and post-processing, limit accessibility, particularly in resource-constrained settings [65]. Finally, this review has focused primarily on T2DM-related DKD given its high prevalence and metabolic complexity. Whether the imaging phenotypes and biomarker thresholds identified here apply equally to T1DM-related nephropathy remains unclear. Elucidating their differing imaging phenotypes remains a critical gap in the field.

Future research should prioritize standardizing cross-institutional acquisition and analysis protocols, validating novel biomarkers and their clinically relevant thresholds in large multi-center cohorts, integrating artificial intelligence for automated, reproducible analysis, and developing targeted molecular imaging probes. These endeavors are crucial for early intervention in DKD, improving risk prediction, and advancing precision medicine.

## 12. Conclusions

Multimodal MRI provides a promising non-invasive approach for evaluating renal structural and functional alterations in DKD. Current evidence supports its potential utility in early disease detection, assessment of pathological heterogeneity, risk stratification, and therapeutic monitoring. Although further standardization and large-scale validation are required before widespread clinical implementation, multiparametric renal MRI may become an important tool for advancing precision medicine in DKD.

## Figures and Tables

**Table 1:** Summary of MRI techniques used in the assessment of pathologic changes in DKD.

Pathological Changes	Techniques	Parameters	Strength	Limitation	References
Renal hypertrophy and volume changes	T1WI/T2WI	TKV (mL), cortical thickness (mm)	High spatial resolution; accurate volumetric assessment; tracks disease progression	Limited tissue characterization; does not detect early functional changes	[8,13]
Renal tissue hypoxia	BOLD	$R_{2}^{*}$, $T_{2}^{*}$	No contrast agent; validated against micropuncture; detects early hypoxia	Influenced by non-oxygenation factors (Hb, hydration, sodium, pH); heterogeneous results in DKD progression; complex analysis	[14,15]
Renal microvascular dysfunction	ASL	Global perfusion (mL/100 g/min), cortex, and medulla perfusion	No exogenous contrast; safe for DKD patients; reproducible	Variation in acquisition schemes; complex post-processing; lower SNR in medulla	[16,17]
	IVIM-DWI	D (mm^2^/s), $D^{*}$, f (%)	Separates perfusion from diffusion; detects capillary impairment in the normoalbuminuric stage	$D^{*}$ and f have lower reproducibility; cardiac cycle fluctuations; inferior to D alone for diagnosis	[18,19,20]
Renal fibrosis or inflammation	DWI/IVIM-DWI	ADC (mm^2^/s), D (mm^2^/s)	Widely available; assesses restricted water diffusion	Affected by perfusion and tubular flow; limited pathological specificity	[18,20,21]
	DTI	FA, MD	Sensitive to medullary structural organization; detects tubular atrophy	Requires multiple gradient directions; technically challenging	[21,22]
	DKI	MK, K_a_, K_r_	Sensitive to non-Gaussian diffusion; detects complex microenvironment	Higher b-values required; computationally intensive	[23,24]
	$T_{1}$ mapping	Native $T_{1}$ (ms), CMD	No contrast needed; reflects ECM expansion and water content; sensitive to tubular injury	Influenced by field strength; non-specific (edema vs. fibrosis)	[25,26]
	MT	MTR (%), PSR	Reflects macromolecular content; correlates with fibrosis in animal models	MTR non-specific; PSR more specific but complex; limited human data	[27,28,29]
Renal steatosis and ectopic fat	Dixon/mDixon Quant	Renal fat fraction (%), PDFF maps	High-resolution fat quantification; separates water and fat; reproducible	Requires dedicated sequences; motion-sensitive; analysis time-consuming	[30,31]
	^1^H-MRS	Renal triglyceride content	Quantifies triglycerides to assess early metabolic abnormalities in the kidney	Sensitive to $B_{0}$ inhomogeneity and respiratory motion; low SNR; complex post-processing	[32,33]
Iron deposition	mDixon Quant/T2WI	$R_{2}^{*}$ (ms), KMR	Detects ferroptosis-related iron overload; routine clinical application possible	Influenced by oxygenation; needs correction for Hb and hydration	[34,35,36]
Sodium metabolism	^23^Na-MRI	Tissue sodium content	Reflects systemic sodium homeostasis; a marker of water-sodium retention	Limited availability; specialized equipment needed; limited human data	[37,38]

T1WI, $T_{1}$-weighted imaging; T2WI, $T_{2}$-weighted imaging; TKV, total kidney volume; BOLD, blood-oxygen-level-dependent; $R_{2}^{*}$, apparent transverse relaxation rate; $T_{2}^{*}$, apparent transverse relaxation time; ASL, arterial spin labeling; SNR, signal-to-noise ratio; IVIM-DWI, intravoxel incoherent motion diffusion-weighted imaging; D, pure diffusion coefficient; $D^{*}$, pseudo-diffusion coefficient; f, perfusion fraction; DWI, diffusion-weighted imaging; MTR, magnetization transfer ratio; ADC, apparent diffusion coefficient; DTI, diffusion tensor imaging; FA, fractional anisotropy; MD, mean diffusivity; DKI, diffusion kurtosis imaging; MK, mean kurtosis; K_a_, axial kurtosis; K_r_, radial kurtosis; CMD, corticomedullary difference; ECM, extracellular matrix; MT, magnetization transfer; MTR, magnetization transfer ratio; PSR, pool size ratio; PDFF, proton density fat fraction; ^1^H-MRS, proton magnetic resonance spectroscopy; KMR, kidney-to-muscle signal intensity ratio; ^23^Na-MRI, sodium-23 magnetic resonance imaging.

**Table 2:** Effects of therapeutic interventions detectable by MRI in DKD.

Drug Class	MRI Technique (Parameter)	Direction of Change	Proposed Mechanism	Representative References
SGLT2 (dapagliflozin)	^23^Na-MRI (tissue sodium content)	Decreased in skin and muscle	Blocking proximal tubular ${Na}^{+}$ reabsorption; correcting systemic water-sodium retention	[147]
GLP-1 RAs (semaglutide)	ASL (RBF)	Increased	Enhanced renal perfusion	[149]
	BOLD-MRI ($R_{2}^{*}$)	Decreased in cortex and medulla	β-Klotho-mediated ferroptosis inhibition; reduced oxidative stress	
	Dixon (FF)	Decreased	Regulation of lipid deposition	
	DTI (FA)	Increased in cortex	Reduced interstitial edema and extracellular matrix remodeling	
GLP-1 RAs (semaglutide/empagliflozin)	DWI (ADC)	Decreased in cortex	Mechanism unclear; changes may be independent of traditional renal function indicators	[150]
ARNI (sacubitril/valsartan)	BOLD-MRI ($R_{2}^{*}$)	Decreased in medulla	Improved renal hemodynamics and metabolic supply-demand balance	[151]
Autologous dendritic cell therapy	DTI (FA)	Increased	Immune microenvironment remodeling; downregulation of inflammatory factors	[152]

## Data Availability

No datasets were generated or analyzed during the current study.

## Abbreviations

ASL	Arterial spin labeling
BOLD	Blood-oxygen-level-dependent
DKD	Diabetic kidney disease
DKI	Diffusion kurtosis imaging
DTI	Diffusion tensor imaging
DWI	Diffusion-weighted imaging
eGFR	Estimated glomerular filtration rate
ESRD	End-stage renal disease
IVIM	Intravoxel incoherent motion
MRI	Magnetic resonance imaging
T2DM	Type 2 diabetes mellitus
UACR	Urinary albumin-to-creatinine ratio
